# Entanglement of Three-Qubit Random Pure States

**DOI:** 10.3390/e20100745

**Published:** 2018-09-29

**Authors:** Marco Enríquez, Francisco Delgado, Karol Życzkowski

**Affiliations:** 1 Escuela de Ingeniería y Ciencias, Tecnológico de Monterrey, Atizapán 52926, Mexico; 2 Faculty of Physics, Astronomy and Applied Computer Science, Jagiellonian University, ul. Łojasiewicza 11, 30-348 Kraków, Poland; 3 Center for Theoretical Physics, Polish Academy of Sciences, Al. Lotników 32/46, 02-668 Warsaw, Poland

**Keywords:** quantum entanglement, three-qubit random states, entanglement classes, entanglement polytope, anisotropic invariants

## Abstract

We study entanglement properties of generic three-qubit pure states. First, we obtain the distributions of both the coefficients and the only phase in the five-term decomposition of Acín et al. for an ensemble of random pure states generated by the Haar measure on U(8). Furthermore, we analyze the probability distributions of two sets of polynomial invariants. One of these sets allows us to classify three-qubit pure states into four classes. Entanglement in each class is characterized using the minimal Rényi-Ingarden-Urbanik entropy. Besides, the fidelity of a three-qubit random state with the closest state in each entanglement class is investigated. We also present a characterization of these classes in terms of the corresponding entanglement polytope. The entanglement classes related to stochastic local operations and classical communication (SLOCC) are analyzed as well from this geometric perspective. The numerical findings suggest some conjectures relating some of those invariants with entanglement properties to be ground in future analytical work.

## 1. Introduction

Entanglement is possibly the most interesting and complex issue in Quantum Mechanics. Due to this phenomenon it is not possible to describe properties of individual subsystems, even though the entire system is known to be in a concrete pure quantum state. Quantification of entanglement is still a challenge for any quantum system consisting of more than two parts [1,2]. The difficulty of the problem grows quickly with the growing number of subsystems and it becomes intractable in the asymptotic limit [3]. Several measures of quantum entanglement were proposed [4], but even in the case of pure states of a multipartite quantum system, it is not possible to identify the single state which can be called the most entangled, as the degree of entanglement depends on the measure used [5].

On the other hand, entanglement in bipartite systems is already well understood. In the case of pure states, a key tool in describing entanglement properties is the Schmidt decomposition as any entanglement measure is a function of the Schmidt coefficients [2]. Dealing with three-party pure states, the problem becomes more intricate as the corresponding state is represented by a tensor rather than a matrix, so one cannot rely on the Schmidt decomposition related to the singular value decomposition of a matrix. Nevertheless, several decompositions for three-qubit states have been studied in literature [6,7,8]. More recently, a canonical form for symmetric three-qubit states has been proposed, showing that in this case the number of entanglement parameters can be reduced from five to three [9].

Early studies on correlation in composite quantum systems revealed that for three or more parties there exist quantum states with different forms of entanglement [8], as the states from one entanglement class cannot be converted by local operations to any states of the other class. As the number of parties increases, the number of entanglement classes grows quickly [10]. Since local operations cannot generate entanglement, one usually assumes that a faithful measure of quantum entanglement should be invariant under local unitary operations and should not grow under arbitrary local operations.

For a given class of operations there exist invariants which are constant along every orbit of equivalent states [11,12]. A full set of invariants determines a given orbit of locally equivalent states. However, such sets of invariants are established only for systems consisting of few parties of a small dimension including the simplest multipartite case of three-qubit systems [13,14,15].

An interesting question arises: To what extent single-particle properties can provide information about the global entanglement [16]? The issue is related to the so-called quantum marginal problem: Given a set of reduced density matrices one asks whether they might appear as partial trace of a given state of a composed system [17]. Necessary conditions for such a “compatibility problem” were provided in [18] for the two-qubit system and then developed by Klyachko [19] for the general case. These conditions can be expressed as a set of linear inequalities concerning the eigenvalues of the density matrix corresponding to the entire system and eigenvalues of the reduced matrices. Interestingly, for multipartite systems the compatibility problem is related to the entanglement characterization [20]. For instance, eigenvalues of three one-qubit reduced matrices of any three-qubit pure state belong to the entanglement polytope and some of its parts correspond to certain classes of quantum entanglement [21].

Not knowing a particular quantum state corresponding to a physical system it is interesting to ask, what are properties of a typical state? More formally, one defines an ensemble of pure quantum states induced by the unitary invariant Fubini-Study measure [2] and computes mean values of various quantities averaging over the unitary group with respect to the Haar measure. Such random quantum states are physically interesting as they arise during time-evolution of quantum systems corresponding to classically chaotic systems [22,23] and are relevant for problems of quantum information processing [24,25].

Research on non-local properties of generic multipartite states has been intensive in recent years. This includes entanglement in two qudit systems [26,27,28], pairwise entanglement in multi-qubit systems [29,30,31], entropic relations and entanglement [32], correlations and fidelities in qutrits system [33], a characterization of entanglement through negativities and tangles in several qubits systems and its relation to the emergence of the bulk geometry [34]. More recently, genuine entanglement for typical states for a system composed out of three subsystems with *d* levels each was studied with help of the geometric measure of entanglement [35], while for generic four-qubit Alsina analyzed the distribution of the hyperdeterminant [36].

The aim of this work is to extend the analysis of entanglement properties of generic states of three-qubit systems. We focus our attention on the five-term decomposition of an arbitrary pure state [15] as it allows one to construct a set of polynomial invariants and to identify the classes of entanglement. We generated an ensemble of pure quantum states induced by the Haar measure on the unitary group U(8) corresponding to the system composed of three qubits and investigated the distribution of various entanglement measures and local invariants.

The paper is organized as follows. In Section 2 we review the five-term decomposition of a three-qubit stateand study statistical properties of the coefficients in such a representation of a generic state. In Section 3, we investigate properties of the three qubits invariants, $I_{k}$ and $J_{k}$ [15] as well as two newly discovered anisotropic invariants [37]. We obtain their probability distributions, either exact or approximate, and compare them with accurate numerical approximations. The fourth section presents an analysis for the entanglement classes defined in terms of the latter invariants. As a comparative element, we use the Rényi and the minimal Rényi-Ingarden-Urbanik (RIU) entropies [35] to analyze possible meanings for such classes. Another measure, the maximum overlap with respect to a selected entanglement class, allows us to identify for an arbitrary three-qubit state the closest state in each class resembling it. In Section 5 we discuss a characterization of quantum entanglement through the corresponding entanglement polytope and we show how entanglement classes can be distinguished from a geometrical viewpoint. The last section presents concluding remarks, a list of open questions with suggestions concerning the future work.

## 2. The Canonical Five-Term Decomposition

A three-qubit state in the Hilbert space $H^{⊗3}$ involves eight terms, thus, it can be written as

(1) $$|\psi \rangle =t^{ijk}|ijk\rangle ,\;t^{ijk}{\bar{t}}_{ijk}=1,\;t^{ijk}\in C,$$

where we have used the repeated scripts notation. It is known [15] that through local unitaries, the number of terms in |ψ〉 can be reduced from eight to five. First, we define the two square matrices $T_{0}$ and $T_{1}$ whose entries are given by ${\left(T_{i}\right)}_{jk}=t^{ijk}$, with i,j,k=0,1. A local unitary transformation $U⊗1_{2}⊗1_{3}$ acting on the first qubit produces

(2) $$T_{0}^{\prime }=u_{00}T_{0}+u_{01}T_{1},\;T_{1}^{\prime }=-{\bar{u}}_{01}T_{0}+{\bar{u}}_{00}T_{1}.$$

The matrix *U* is taken such that $\det(T_{0}^{\prime })=0$. On the other hand, the transformation $1_{2}⊗V⊗W$ changes the matrices $T_{i}$ according to $VT_{i}W$. We choose *V* and *W* so that $T_{0}^{\prime }$ can be diagonalized via the singular value decomposition (SVD). Explicitly, at the end of this procedure we arrive at

(3) $$T_{0}^{\prime \prime }=\left(\begin{array}{cc} \lambda_{0} & 0\\ 0 & 0 \end{array}\right),\;\;T_{1}^{\prime \prime }=\left(\begin{array}{cc} {\tilde{\lambda }}_{1} & {\tilde{\lambda }}_{2}\\ {\tilde{\lambda }}_{3} & {\tilde{\lambda }}_{4} \end{array}\right).$$

In addition, the phase of the coefficients ${\tilde{\lambda }}_{2},{\tilde{\lambda }}_{3}$ and ${\tilde{\lambda }}_{4}$ can be absorbed into ${\tilde{\lambda }}_{1}$ to yield the decomposition

(4) $$|\psi \rangle =\lambda_{0}|000\rangle +\lambda_{1}e^{i\phi }|100\rangle +\lambda_{2}|101\rangle +\lambda_{3}|110\rangle +\lambda_{4}|111\rangle ,$$

where $\lambda_{i},\phi \in R$. Besides $\sum \lambda_{i}^{2}=1$. According to [15], the only phase ϕ should be restricted to 0<ϕ<π to assure the uniqueness of the decomposition.

### Distribution of the Coefficients

**Figure 1:**
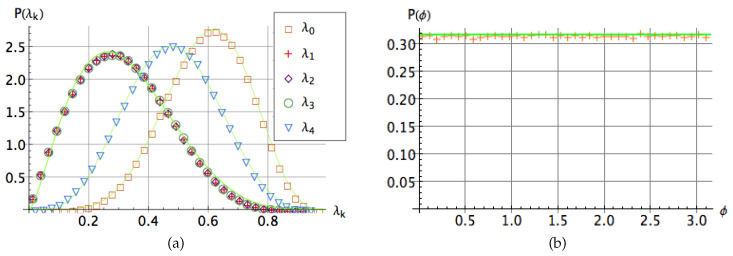
Probability distribution of the Acín parameters in the state (4): (**a**) the coefficients $\lambda_{k},k=0,1,\ldots ,4$ and (**b**) the phase ϕ for a set of $10^{6}$ three-qubit random states on $H_{2}^{⊗3}$. Solid lines represent the best numerical fit in all the cases, the parameters of which are listed in Table 1.

We take an ensemble of $10^{6}$ random states in $H^{⊗3}$ distributed according to the unitary invariant measure on the group U(8) and then first reduce them into the five-term representation (4), then we track each coefficient $\lambda_{k}$ to compute numerically its probability distributions as well as the distribution of the phase ϕ. The result is shown in Figure 1 depicting the value of each component $\lambda_{k}$ versus their relative normalized density on $H^{⊗3}$. Note that the state (1) depends on 14 real parameters, say $p=(p_{1},\ldots ,p_{14})$ where each $p_{\mu }$ is the real or imaginary part of $t_{ijk}$. The unitary invariance implies that after the action of the transformation U⊗V⊗W on the state |ψ〉 the distribution of the coefficients $\lambda_{i}$’s and the phase ϕ fulfils P(p)=J×P(λ), where $\lambda =(\lambda_{0},\lambda_{1},\lambda_{2},\lambda_{3},\lambda_{4},\phi )$ and *J* is the Jacobian of the transformation. The evaluation of this 14×14 determinant becomes cumbersome and one has to rely on numerical methods to compute the marginal distributions $P(\lambda_{k})$ of the coefficients of the state (4) as well as the phase ϕ. The data presented in Figure 1b suggest that the phase ϕ is distributed uniformly on the entire range, P(ϕ)=1/π for ϕ∈[0,π]. As the beta distribution has been used to model the behavior of random variables limited to finite length intervals in several contexts [24,35,38], we propose the following distribution $P_{i}\left(\lambda_{i}\right)=c\;\lambda_{i}^{a}{\left(1-\lambda_{i}\right)}^{b}$, to fit the distributions of the coefficients $\lambda_{j}$. The numerical fits are depicted as solid lines in Figure 1a and the values of the best fitting parameters are reported in Table 1. Results presented suggest that the coefficients $\lambda_{1}$, $\lambda_{2}$ and $\lambda_{3}$ are distributed according to the same probability distribution. Hence, we conjecture that out of the six real parameters in Equation (4), only four are required to characterize entanglement in three-qubit random states, say $\left\{\lambda_{0},\lambda_{1},\lambda_{4},\phi \right\}$. Interestingly, the coefficients $\lambda_{0}$ and $\lambda_{4}$ are related with the invariant $J_{4}$ connected with the three-qubit genuine entanglement (for the definition see subsequent section). As generic three-qubit states are typically strongly entangled [35], this analysis illustrates how each coefficient $\lambda_{j}$ of a given state is linked with the degree of its entanglement. Note particularly how low values of $\lambda_{1},\lambda_{2}$ and $\lambda_{3}$ are more representative for entangled states in contrast to $\lambda_{4}$, the distribution of which appears to be balanced. Furthermore, the higher values of the coefficient $\lambda_{0}$ correspond to the states with larger entanglement. This is particularly interesting as in the decomposition of Carteret et al. this coefficient yields the maximum overlap with the closest separable state [7].

## 3. Three-Qubits Polynomial Invariants

Local unitary (LU) transformations performed on individual subsystems define orbits of locally equivalent multipartite states. Local invariants can be understood as coordinates in the space of orbits of locally equivalent states. Any complete set of local invariants allows one to distinguish between different orbits of locally equivalent states and thus to describe the degree of quantum entanglement [7]. For pure states of a three-qubit system, the space of orbits has six dimensions and it is possible to find six algebraically independent invariants [39].

In this section we will analyze the distributions $P(I_{k})$ and $P(J_{k})$ on $H^{⊗3}$ for the corresponding three-qubit invariants (under local operations) $I_{k}$ [13] and $J_{k}$ [40], with k=1,…,5. These polynomial invariants set representative classes on $H^{⊗3}$ and cannot be directly used as the measures of genuine entanglement.

### Distribution of the Invariants

We first consider the set of five invariants used in [40]

(5) $$\begin{array}{c} I_{2}=\mathrm{tr}\left(\rho_{A}^{2}\right),\;I_{3}=\mathrm{tr}\left(\rho_{B}^{2}\right),\;I_{4}=\mathrm{tr}\left(\rho_{C}^{2}\right),\\ I_{5}^{\prime \prime \prime }=\mathrm{tr}\left[\left(\rho_{A}⊗\rho_{B}\right)\rho_{AB}\right],\;I_{6}={\left|\mathrm{Hdet}\left(T\right)\right|}^{2} \end{array}$$

where $\rho_{i}$ stands for the reduced density matrix of the *i*-th system, $\rho_{ij}$ is the reduced density matrix when the partial trace respect the system *k* is performed while i,j,k is a permutation of A,B,C. The last invariant is related to the hyperdeterminant Hdet of the tensor coefficients $T=(t^{ijk})$ representing the state (1).

**Figure 2:**
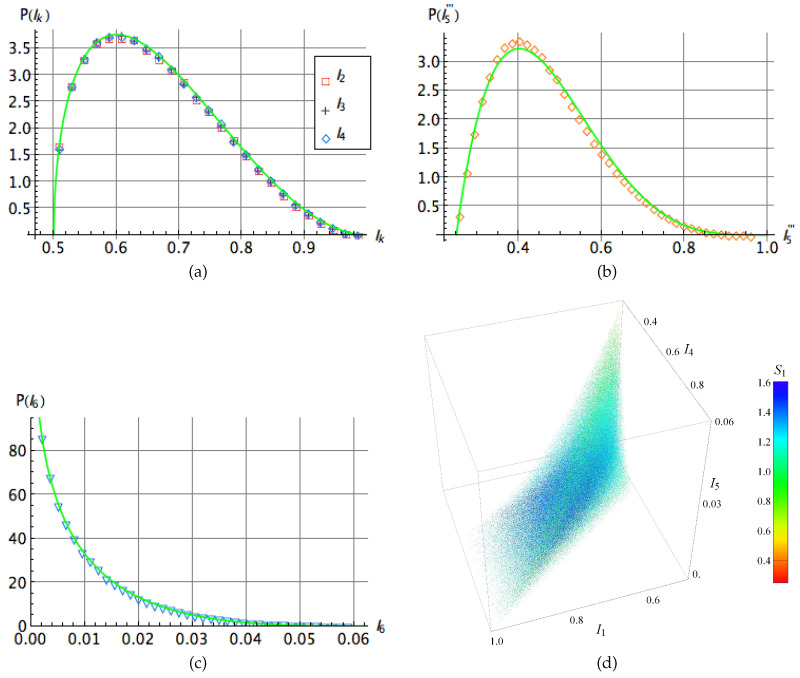
(**a**–**c**) Probability distribution for the polynomial invariants $I_{i},i=1,\ldots ,5$ for a set of $10^{6}$ three-qubit random states. Solid line in panel (**a**) stands for the distribution (9), while in panels (**b**,**c**) the best numerical distributions are depicted by green curves. In panel (**d**) a dispersion plot comparing $I_{1},I_{4}$ and $I_{5}$ is shown. In addition, each dot has been colored as function of its $S_{1}$ Rényi entropy [41] calculated after the five terms reduction.

The invariants are labeled according to the notation used by Sudbery [13]. Note that the squared norm of the state (1) is in itself a polynomial invariant usually denoted as $I_{1}$. In Figure 2a–c we show the probability distribution of the above set of invariants over an ensemble of $10^{6}$ random states. Moreover, as for k=2,3,4 the quantity $I_{k}$ is related with the linear entropy, $S_{k}=1-I_{k}$, the corresponding distributions show that the entanglement of each qubit with the other two is the same no matter which partial trace is performed. On the other hand, the invariants $I_{k}$ in terms of the coefficients $t^{ijk}$ are written as [13]:

(6) $$\begin{array}{c} I_{2}=t^{i_{1}j_{1}k_{1}}{\bar{t}}_{i_{2}j_{1}k_{1}}t^{i_{2}j_{2}k_{2}}{\bar{t}}_{i_{1}j_{2}k_{2}},\;I_{3}=t^{i_{1}j_{1}k_{1}}{\bar{t}}_{i_{1}j_{2}k_{1}}t^{i_{2}j_{2}k_{2}}{\bar{t}}_{i_{2}j_{1}k_{2}},\;I_{4}=t^{i_{1}j_{1}k_{1}}{\bar{t}}_{i_{1}j_{1}k_{2}}t^{i_{2}j_{2}k_{2}}{\bar{t}}_{i_{2}j_{2}k_{1}},\\ I_{5}^{\prime \prime \prime }=t^{i_{1}j_{1}k_{1}}{\bar{t}}_{i_{1}j_{2}k_{2}}t^{i_{2}j_{2}k_{2}}{\bar{t}}_{i_{2}j_{3}k_{1}}t^{i_{3}j_{3}k_{3}}{\bar{t}}_{i_{3}j_{1}k_{3}}\\ I_{6}=4{\left|\epsilon_{i_{1},j_{1}}\epsilon_{i_{2}j_{2}}\epsilon_{k_{1}\ell_{1}}\epsilon_{k_{2}\ell_{2}}\epsilon_{i_{3}k_{3}}\epsilon_{j_{3}\ell_{3}}t^{i_{1}i_{2}i_{3}}t^{j_{1}j_{2}j_{3}}t^{k_{1}k_{2}k_{3}}t^{\ell_{1}\ell_{2}\ell_{3}}\right|}^{2} \end{array}$$

where the convention of summation over repeated indexes is used and $\epsilon_{k,\ell }$ stands for the Levi-Civita tensor of order two. Since the coefficients can be regarded as a column of a random unitary matrix, we can compute the average value of each invariant by evaluating integrals of polynomial functions over the unitary group with respect to unique normalized Haar measure. Using symbolic integration [42] we obtain $\langle I_{k}\rangle =2/3$ for k=2,3,4. This result is consistent with the mean purity of a single qubit traced out from a 2×4 system reported in [43]. Moreover, $\langle I_{5}^{\prime \prime \prime }\rangle =7/15$ and $\langle I_{5}^{{}^{\prime \prime \prime }2}\rangle =133/572$. In order to compute the mean value of $I_{6}$, we use the second moment of the three-tangle τ reported in [35] with the fact $\tau^{2}=16I_{6}$ to get $\langle I_{6}\rangle =1/110$. On the other hand, to compute the distributions of the invariants $P(I_{k})$ for k=2,3,4, we first note that the joint density of eigenvalues $\vartheta_{1}$ and $\vartheta_{2}$ of a single qubit traced out of a system of a three-qubit system is given in Equation (6) of [43] with N=2 and K=4. This reads

(7) $$P\left(\vartheta_{1},\vartheta_{2}\right)=210\;\delta \left(1-\vartheta_{1}-\vartheta_{2}\right){\left(\vartheta_{1}-\vartheta_{2}\right)}^{2}\vartheta_{1}^{2}\vartheta_{2}^{2}$$

where δ stands for the Dirac delta. As each $I_{k}$ is nothing other than the purity of a single qubit reduced density matrix, we can compute the probability distribution by performing the following integral

(8) $$P\left(I_{k}\right)=210\int_{0}^{1}\int_{0}^{1}d\vartheta_{1}d\vartheta_{2}P\left(\vartheta_{1},\vartheta_{2}\right)\delta \left(I_{k}-\vartheta_{1}^{2}-\vartheta_{2}^{2}\right),$$

this yields

(9) $$P\left(I_{k}\right)=\frac{105}{2}{\left(1-I_{k}\right)}^{2}{\left(2I_{k}-1\right)}^{1/2},\;1/2\leq I_{k}\leq 1,\;k=2,3,4.$$

This probability distribution is depicted in Figure 2. In addition, we approximate the distribution $P(I_{5}^{\prime \prime \prime })$ by the following beta distribution

(10) $$P_{F_{5}}\left(I_{5}^{\prime \prime \prime }\right)=\frac{\Gamma (a+b+2)}{3^{a+b+1}4^{a+1}\Gamma \left(a+1\right)\Gamma \left(b+1\right)}{\left(1-I_{5}^{\prime \prime \prime }\right)}^{a}{\left(4I_{5}^{\prime \prime \prime }-1\right)}^{b},$$

requiring the first two moments of this distribution coincide with the exact two moments of $P(I_{5}^{\prime \prime \prime })$ reported above. We found a=21,989/5691 and b=5554/5691. On the other hand, the distribution of the square of the three tangle was approximated in [35] by a Beta distribution. Thus, making a variable change in this result we may approximate $P(I_{6})$ by

(11) $$P_{F_{6}}\left(I_{6}\right)=\frac{2}{\sqrt{I_{6}}}\mathrm{Beta}\left(31/17,62/17,4\sqrt{I_{6}}\right),\;0\leq I_{6}\leq 1/16.$$

As the distributions of the invariants $I_{2},I_{3}$ and $I_{4}$ are the same, we only need three invariants to characterize the entanglement in the set of three-qubit random states, say $(I_{2},I_{5}^{\prime \prime \prime },I_{6})$. In Figure 2d we show a dispersion plot whose three axes correspond to such invariants and their colors correspond to their $S_{1}$ Rényi entropy calculated after of the five terms reduction [35] (which will be properly presented in the next section) in agreement with the side color scale.

**Figure 3:**
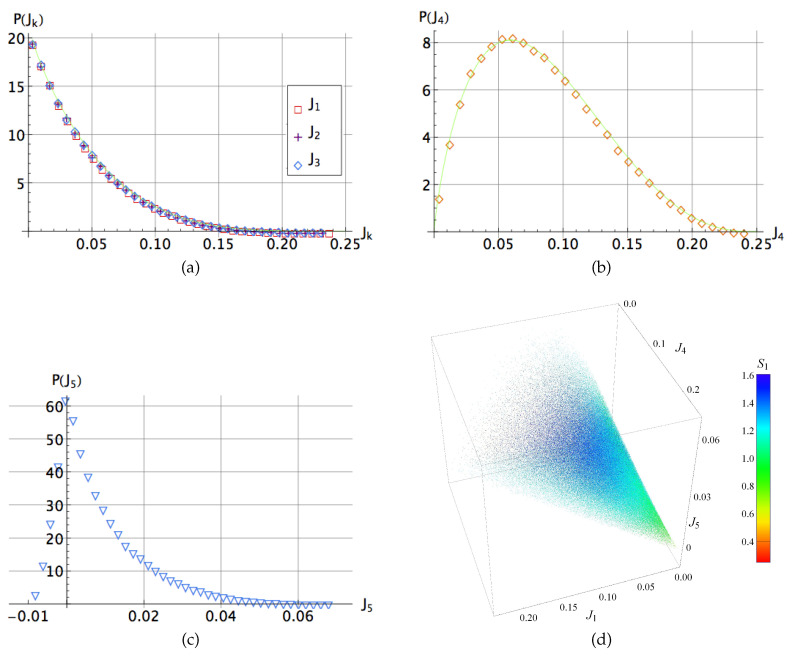
(**a**–**c**) Probability distribution for the polynomial invariants $J_{i},i=1,\ldots ,5$ for a set of $10^{6}$ three-qubit random states. In all graphics the numerical best fit distribution is depicted as the green line. In Figure (**d**) we show a dispersion plot comparing $J_{1},J_{4}$ and $J_{5}$. In addition, each dot has been colored as a function of its $S_{1}$ Rényi entropy [41] calculated after the five term reduction in agreement with the side color scale.

We also consider the set of invariants proposed by Acín et al. [15]. These invariants allow to identify different entanglement classes (which will be discussed in the next section) and can be written in terms of the six parameters of the five-term decomposition as

(12) $$\begin{array}{c} J_{1}={\left|\lambda_{1}\lambda_{4}e^{i\varphi }-\lambda_{2}\lambda_{3}\right|}^{2},\;J_{2}=\mu_{0}\mu_{2},\;J_{3}=\mu_{0}\mu_{3},\\ J_{4}=\mu_{0}\mu_{4},\;J_{5}=\mu_{0}\left(J_{1}+\mu_{2}\mu_{3}-\mu_{1}\mu_{4}\right), \end{array}$$

where $\mu_{i}=\lambda_{i}^{2}$. For this analysis, the same set of $10^{6}$ random states was considered but they are now used to obtain the corresponding values of them through their expressions in terms of the five-term coefficients [15]. All these invariants can be calculated departing from the set of $\lambda_{i}$. The outcomes are shown in the Figure 2 and Figure 3 in their respective ranges. Note in the Figure 3a–c how for $J_{1},J_{2}$ and $J_{3}$ the distribution is biased on low values of these invariants, denoting a possible relation with higher entanglement. For the quantity $J_{4}$, related to the hyperdeterminant, the distribution peaks around of $\frac{1}{16}$, denoting that separability as well as genuine entanglement are absent in the most of states in $H^{⊗3}$. A similar feature is observed for $J_{5}$ but varying sharply for negative and positive values. On the other hand, the invariants $J_{k}$’s can be expressed in terms of the quantities $I_{k}$’s [15]

$$\begin{array}{c} J_{1}=\frac{1}{4}\left(1+I_{2}-I_{3}-I_{4}-2\sqrt{I_{6}}\right),\;J_{2}=\frac{1}{4}\left(1-I_{2}+I_{3}-I_{4}-2\sqrt{I_{6}}\right),\\ J_{3}=\frac{1}{4}\left(1-I_{2}-I_{3}+I_{4}-2\sqrt{I_{6}}\right),\;J_{4}=\sqrt{I_{6}},\\ J_{5}=\frac{1}{4}\left(3-3I_{2}-3I_{3}-I_{4}+4I_{5}-2\sqrt{I_{6}}\right). \end{array}$$

Such expressions are useful to compute some averages. For instance, as $\langle \sqrt{I_{5}}\rangle =\langle \tau \rangle /4$ it is immediate to compute $\langle J_{4}\rangle =1/12$. From the above definitions we can calculate directly $\langle J_{k}\rangle =1/24$, for k=1,2,3 and $\langle J_{5}\rangle =1/120$. We approximate the probability distributions $P(J_{k})$ with k=1,2,3 by a distribution $P_{F_{k}}\left(J_{k}\right)∼J_{k}^{a}{\left(1-4J_{k}\right)}^{b}$, where the parameters in this case are determined numerically to yield the best fit. In addition, making use of the approximation (11) for the distribution of the invariant $I_{6}$. One can obtain the following approximation for the distribution of the variable $J_{4}$

(13) $$P_{F4}\left(J_{4}\right)=4\mathrm{Beta}\left(31/17,62/17;4J_{4}\right),\;0\leq J_{4}\leq 1/4$$

On the other hand, as the distributions for $J_{1},$$J_{2}$ and $J_{4}$ are uniform among them, we may characterize the entanglement using only the invariants $J_{1},J_{4}$ and $J_{5}$. In Figure 3d we depict a scatter plot using these invariants as coordinates, similarly as in Figure 2d for $I_{k}$.

Another interesting invariant is the one obtained by Kempe [44]

(14) $$I_{5}=3\mathrm{tr}\left(\rho_{A}⊗\rho_{B}\right)\rho_{AB}-\mathrm{tr}\rho_{A}^{3}-\mathrm{tr}\rho_{B}^{3}=t^{i_{1}j_{1}k_{1}}t^{i_{2}j_{2}k_{2}}t^{i_{3}j_{3}k_{3}}{\bar{t}}_{i_{1}j_{2}k_{3}}{\bar{t}}_{i_{2}j_{3}k_{1}}{\bar{t}}_{i_{3}j_{1}k_{2}},$$

which distinguishes locally indistinguishable states. In terms of the Acín parameters, it reads

(15) $$\begin{array}{cc} I_{5}= & 1-3\lambda_{4}^{2}-3\lambda_{3}^{2}+3\lambda_{3}^{4}+3\lambda_{4}^{4}+3\lambda_{1}^{2}\lambda_{3}^{2}+6\lambda_{3}^{2}\lambda_{4}^{2}\\ & \;+\left(\lambda_{1}^{2}\left(3-6\lambda_{3}^{2}\right)-3\left(\lambda_{3}^{2}-1\right)\left(2\lambda_{3}^{2}+2\lambda_{4}^{2}-1\right)\right)\lambda_{2}^{2}\\ & \;+6\lambda_{1}\lambda_{3}\lambda_{4}\left(\lambda_{1}^{2}+\lambda_{2}^{2}+\lambda_{3}^{2}+\lambda_{4}^{2}\right)\lambda_{2}\cos\phi +\left(3-6\lambda_{3}^{2}\right)\lambda_{2}^{4}. \end{array}$$

**Figure 4:**
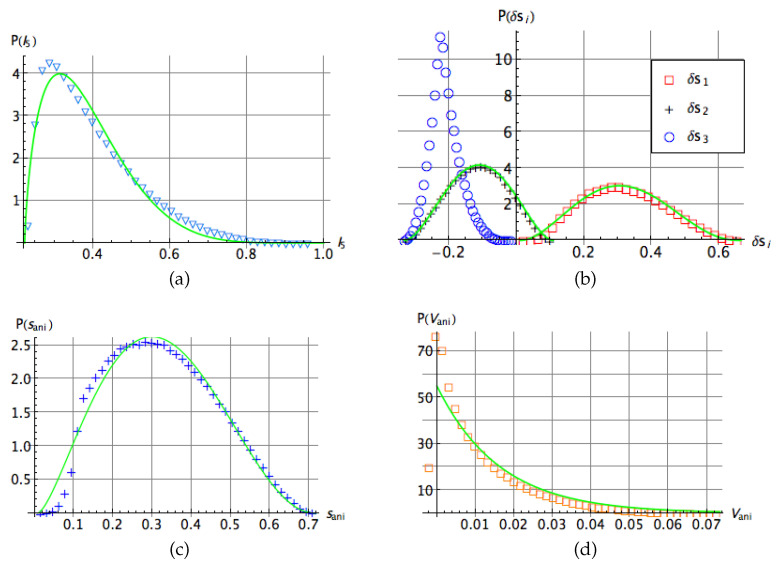
Probability distribution of: (**a**) the Kempe invariant $I_{5}$ (the green line stands for the probability distribution (16)). (**b**) The pairwise anisotropic strengths $\delta s_{j}$ with j=1,2,3. (**c**) The invariant $s_{\mathrm{ani}}$ and (**d**) The invariant $V_{\mathrm{ani}}$. Solid lines in all cases correspond to the best numerical fit.

Note that the form (14) of the Kempe Invariant $I_{5}$ is manifestly permutation symmetric. Although this quantity cannot be considered as a legitimate measure of entanglement, Osterloh has pointed out [45] that different values of $I_{5}$ allow to distinguish between different local orbits of three qubit pure states. Integrating Equation (14) using symbolic integration on the Haar measure, we found that $\langle I_{5}\rangle =2/5$ and $\langle I_{5}^{2}\rangle =499/2860$. In Figure 4a, we show the probability distribution of the invariant $I_{5}$, which can be approximated by the distribution

(16) $$P_{F_{I_{5}}}\left(\kappa \right)=\frac{9^{a+1}\Gamma \left(a+b+2\right)}{7^{a+b+1}\Gamma \left(a+1\right)\Gamma \left(b+1\right)}{\left(1-\kappa \right)}^{a}{\left(9\kappa -2\right)}^{b},\;2/9\leq \kappa \leq 1$$

where a=90/23 and b=283/621 are settled by the condition that the first two moments of $P_{F_{I_{5}}}\left(I_{5}\right)$ correspond with the first two moments of $P(I_{5})$ provided above. We remark that sextic invariant $I_{5}^{\prime \prime \prime }$ can be written in terms of the Kempe invariant and the quadratic and quartic invariants [13].

Recently, an alternative set of invariants characterizing a three-qubit pure state |ψ〉 was proposed by Cheng and Hall [37]. To define them, consider a two-qubit reduced density matrix $\rho^{kl}={\mathrm{Tr}}_{m}|\psi_{klm}\rangle \langle \psi_{klm}|$ where indices k,l,m denote three subsystems A,B,C and m≠k,l. Any such a matrix of order four can be written in its Bloch representation,

(17) $$\begin{array}{c} \rho^{kl}=\frac{1}{4}\left(1^{k}⊗1^{l}+K\cdot {\vec{\sigma }}^{k}⊗1^{l}+1^{k}⊗L\cdot {\vec{\sigma }}^{l}+\sum_{i,j=1}^{3}T_{i,j}^{k,l}\sigma_{i}^{k}⊗\sigma_{j}^{l}\right) \end{array}$$

where ${\vec{\sigma }}^{k}=\left(\sigma_{1}^{k},\sigma_{2}^{k},\sigma_{3}^{k}\right)$, while K and L denote the Bloch vectors for parts *k* and *l* respectively. Entries of the correlation matrices of the reduced states read $T_{n,m}^{k,l}=\langle \sigma_{n}^{k}⊗\sigma_{m}^{l}\rangle =\mathrm{Tr}\left(\sigma_{n}^{k}⊗\sigma_{m}^{l}\rho^{kl}\right)$, while the superscripts denote two out of three subsystems A,B,C as required to determine a two-qubit partial trace. Let $s_{j}^{kl}$ denote the eigenvalues of the symmetric matrix $S^{kl}=T^{kl}{\left(T^{kl}\right)}^{†}$ and the average value read, $s_{\mathrm{iso}}^{kl}=\left(s_{1}^{kl}+s_{2}^{kl}+s_{3}^{kl}\right)/3$. The invariants are constructed in terms of the pairwise anisotropic strengths $\delta s_{j}^{AB}$, $\delta s_{j}^{AC}$ and $\delta s_{j}^{BC}$ with j=1,2,3 which read $\delta s_{j}^{kl}=s_{j}^{kl}-s_{\mathrm{iso}}^{kl}$, with k,l=A,B,C. It was shown [37] that the pairwise anisotropic strengths fulfil the relations

(18) $$\delta s_{j}=\delta s_{j}^{AB}=\delta s_{j}^{AC}=\delta s_{j}^{BC},\;j=1,2,3$$

and they are also invariant under local transformations as well as any permutation of the parties. Hence, the anisotropic strength and the anisotropic volume can be defined as

(19) $$s_{\mathrm{ani}}^{2}=\sum_{i}{\left(\delta s_{i}\right)}^{2},\;V_{\mathrm{ani}}=\prod_{j}\delta s_{i}.$$

Note that for a given three-qubit pure state |ψ〉 the above invariants can be related with parameters entering the five-term form (4) —see Supplementary Material in [37].

In Figure 4b–d we show the probability distributions of the pairwise anisotropic strengths as well as the probability distribution of the invariants $s_{\mathrm{ani}}$ and $V_{\mathrm{ani}}$ for an ensemble of $10^{6}$ three-qubit random states. We approximate numerically the distribution of the quantities $\delta s_{1}$, $\delta s_{2}$ and $s_{\mathrm{ani}}$ by respective beta distributions:

(20) $$\begin{array}{c} P_{F}\left(\delta s_{1}\right)=c_{1}{\left(a_{1}-\delta s_{1}\right)}^{\beta_{1}}{\left(\delta s_{1}-a_{2}\right)}^{\beta_{2}},\;P_{F}\left(\delta s_{2}\right)=c_{2}{\left(a_{3}-\delta s_{2}\right)}^{\beta_{3}}{\left(a_{4}+\delta s_{2}\right)}^{\beta_{4}},\\ P_{F}\left(s_{\mathrm{ani}}\right)=c_{3}{\left(a_{5}-s_{\mathrm{ani}}\right)}^{\beta_{5}}s_{\mathrm{ani}}^{\beta_{6}}, \end{array}$$

while the positive part of the distribution of $V_{}$ can be approximated by an exponential distribution,

(21) $$P_{F}\left(V_{\mathrm{ani}}\right)=c_{4}e^{-bV_{\mathrm{ani}}}.$$

The fitting parameters read $c_{i}=\left(472.7,1299,135.6,54.9\right)$ for i=1,…,4; $a_{j}=\left(0.66,0.01,0.11,0.33,0.72\right)$ for j=1,…,5; $\beta_{i}=\left(2.5,2.04,1.88,1.92,2.26,1.63\right)$ for i=1,…,6 and b≈61.6. Interestingly, the distribution of the negative quantity $\delta s_{3}$ displays a singular peak, while the distribution of $V_{\mathrm{ani}}$ attains its maximum at anisotropic volume close to zero and exhibits an exponential decay.

## 4. Three-Qubits Entanglement Classes

A state classification has been presented in [15] based on the minimal number of product states in (4). Acín et al. reported some entanglement classes which are presented in Table 2. The conditions for such class states are expressed in terms of the invariants $J_{k}$. Thus, in this section we consider the invariant classes introduced there, departing from the coefficients of the five-term representation in $H^{⊗3}$. These classes barely describe some families around some characteristic states in this space. The first aim is to analyze how those classes represent the entanglement of each state included there, mainly based on the entanglement distribution knowledge on $H^{⊗3}$ [35]. Note that in some classes the direct imposition of the conditions on the invariants leaves some product states that differ from those reported by Acín, that is to say, to obtain such product states an additional LU transformation is required. Such cases are remarked with ⋆ in Table 2.

### 4.1. The Minimal Decomposition Entropy

We characterize the entanglement degree of the classes in Table 2 using the minimal Rényi-Ingarden-Urbanik (RIU) entropy, also known as minimal decomposition entropy [35]. For the state (1) this is defined as

(22) $$S_{q}^{\mathrm{RIU}}\left(\psi \right)\;:=\;\min_{U_{\mathrm{loc}}}S_{q}\left[p(U_{\mathrm{loc}}|\psi \rangle )\right],$$

where p(·) stands for the probability vector related to the state (1) and the minimum is taken on all local transformations $U_{\mathrm{loc}}=U_{1}⊗U_{2}⊗U_{3}$. Note that $S_{q}$ is the *q*-order Rényi entropy [41]. Depending on the parameter *q* the quantity (22) provides information about the state [35]. Thus, for

- q=0: The decomposition entropy is related to the tensor rank of the state |ψ〉. As a direct consequence of the decomposition (4) we have $S_{0}^{\mathrm{RIU}}\left(\psi \right)\leq 5$.
- q=1: The minimal decomposition entropy $S_{1}^{\mathrm{RIU}}(|\psi \rangle )$ determines the minimal information gained by the environment after performing a projective von-Neumann measurement of the pure state |ψ〉〈ψ| in an arbitrary product basis [46].
- q→∞: In such a limiting case, the minimal RIU entropy is associated with the maximal overlap with the closest separable state $\Lambda_{\max}=\max{\left|\langle \psi |\chi_{\mathrm{sep}}\rangle \right|}^{2}$. Indeed, it can be shown that $S_{\infty }^{\mathrm{RIU}}(|\psi \rangle )=-\log\lambda_{\max}$. See [35] for details.

A direct computation shows that for a state in class 1, the minimal RIU entropy vanishes regardless of the value of the parameter *q*. The corresponding calculation for the other entanglement classes is presented below.

#### 4.1.1. Classes 2

A direct calculation shows that the decomposition of states in class 2b is optimal. That is to say, if the state is given by

(23) $$|\varphi_{2b}\rangle =\cos\alpha |000\rangle +\sin\alpha |111\rangle ,\;0<\alpha <\pi /2,$$

the minimal decomposition entropy reads

(24) $$S_{1}^{\mathrm{RIU}}\left(\varphi_{2b}\right)=-\cos^{2}\alpha \ln\left(\cos^{2}\alpha \right)-\sin^{2}\alpha \ln\left(\sin^{2}\alpha \right).$$

Our numeric calculations indicate that for the class 2a, the Acín decomposition is optimal as well. The states with the largest minimal decomposition entropy in each class are

(25) $$|\varphi_{2a}^{\max}\rangle =\frac{1}{\sqrt{2}}|000\rangle +\frac{1}{\sqrt{2}}\left|111\rangle ,\;\right|\varphi_{2b}^{\max}\rangle =\frac{1}{\sqrt{2}}|100\rangle +\frac{1}{\sqrt{2}}|111\rangle ,$$

note the reported basis for class 2b in Table 2 is different due to additional changes commonly reported in the literature. A simple calculation shows the LU equivalence of the two local basis. Note that the state $|\varphi_{2b}^{\max}\rangle$ is bi-separable and it attains the same minimal decomposition entropy as the GHZ state.

#### 4.1.2. Classes 3

Any state belonging to class 3a can be parametrized as

(26) $$|\varphi_{3a}\rangle =\sin\theta_{1}\sin\theta_{2}|000\rangle +\sin\theta_{1}\cos\theta_{2}|101\rangle +\cos\theta_{1}|110\rangle ,\;0<\theta_{1},\theta_{2}<\pi /2$$

note such state is LU-equivalent to the symmetric state

(27) $$|{\tilde{\varphi }}_{3a}\rangle =\sin\theta_{1}\sin\theta_{2}|100\rangle +\sin\theta_{1}\cos\theta_{2}|001\rangle +\cos\theta_{1}|010\rangle ,$$

hence, the minimal RIU entropy can be computed using the method described in [35] for symmetric states. In particular, if $\cos\theta_{1}=1/\sqrt{3}$ and $\sin\theta_{2}=1/\sqrt{2}$ we obtain the well-known *W*-state for which $S_{1}^{\mathrm{RIU}}\left(W\right)=\ln3$, which is the largest value of $S_{1}^{\mathrm{RIU}}$ for this class.

On the other hand, a state in class 3b can be written as

(28) $$|\varphi_{3b}\rangle =\sin\theta_{1}\sin\theta_{2}|000\rangle +\sin\theta_{1}\cos\theta_{2}|110\rangle +\cos\theta_{1}|111\rangle ,\;0<\theta_{1},\theta_{2}<\pi /2.$$

**Figure 5:**
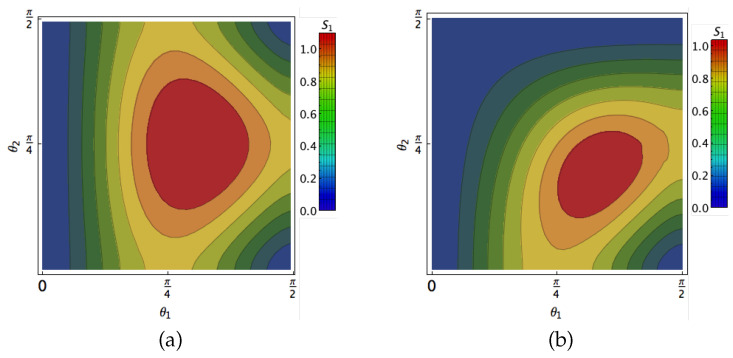
(**a**) The minimal decomposition entropy level curves as function of the parameters $\theta_{1}$ and $\theta_{2}$ for a state in class 3a; (**b**) Same as (**a**) for a state in class 3b.

No state in class 3b has greater $S_{1}^{\mathrm{RIU}}$ than the *W*-state. For a general state in these classes, the minimal decomposition entropy as a function of parameters $\theta_{1}$ and $\theta_{2}$ is depicted in Figure 5. Note that regions of maximal $S_{1}^{\mathrm{RIU}}$ entropy are around the values $\theta_{1},\theta_{2}$ for the maximal entropy for such states.

#### 4.1.3. Classes 4

**Figure 6:**
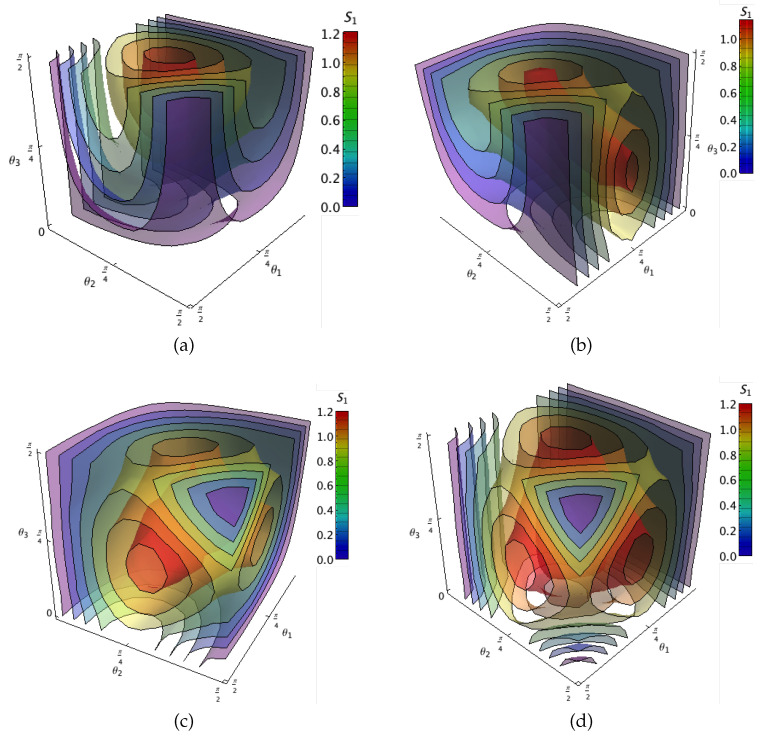
Surfaces of equal entanglement for classes 4 measured with respect the minimal decomposition entropy as function of the parameters $\theta_{1}$, $\theta_{2}$ and $\theta_{3}$ defining each class. Different panels correspond to a state in: (**a**) Class 4a; (**b**) Class 4b; (**c**) Class 4c; (**d**) Class 4d.

A general state in each one of the classes 4 can be written as

(29) $$\begin{array}{ccc} |\varphi_{4a}\rangle & = & \beta_{1}|000\rangle +e^{i\varphi }\beta_{2}|100\rangle +\beta_{3}|101\rangle +\beta_{4}|110\rangle \end{array}$$

(30) $$\begin{array}{ccc} |\varphi_{4b}\rangle & = & \beta_{1}|000\rangle +e^{i\varphi }\beta_{2}|100\rangle +\beta_{3}|110\rangle +\beta_{4}|111\rangle \end{array}$$

(31) $$\begin{array}{ccc} |\varphi_{4c}\rangle & = & \beta_{1}|000\rangle +\beta_{2}|101\rangle +\beta_{3}|110\rangle +\beta_{4}|111\rangle \end{array}$$

(32) $$\begin{array}{ccc} |\varphi_{4d}\rangle & = & \beta_{1}|000\rangle +\beta_{2}|010\rangle +\beta_{3}|100\rangle +\beta_{4}|111\rangle \end{array}$$

where $\beta_{1}=\sin\theta_{1}\sin\theta_{2}\sin\theta_{3},\beta_{2}=\sin\theta_{1}\sin\theta_{2}\cos\theta_{3},\beta_{3}=\sin\theta_{1}\cos\theta_{2}$ and $\beta_{4}=\cos\theta_{1}$. As for class 2b, the basis elements for class 4d reported in Table 2 are not those directly obtained from (4). Class 4d corresponds to the real class (with all components real, thus $e^{i\varphi }=\pm 1$) which allows for the performance of an additional reduction to only four terms. As in the previous case, we get the surfaces of minimal decomposition entropy in terms of parameters $\theta_{1},\theta_{2}$ and $\theta_{3}$ in the Figure 6. Those figures exhibit for each class the behavior for the entropy. There, the frontiers of the regions shown $\theta_{1},\theta_{2},\theta_{3}=0,\pi /2$ correspond to separable states. In addition, our numerical calculations show that the minimal decomposition entropy is independent of the phase ϕ. We also numerically found that the the largest $S_{1}^{\mathrm{RIU}}\left(\psi_{4a}^{\max}\right)=1.213$ is attained for a state in class 4a with $\theta_{1}=3\pi /10$, $\theta_{2}=4\pi /15$ and $\theta_{3}=23\pi /60$. Note that this value is smaller than the one reported earlier [35] as the maximal for a random state with five components.

### 4.2. The Maximum Overlap with an Entanglement Class

Given an ensemble of random states, a natural question arises: how many states of such ensemble belong to a particular Acín entanglement class? To tackle this question, observe first that numeric calculations imply $\langle S_{0}^{\mathrm{RIU}}\left(\psi \right)\rangle =\log5$. Hence a generic three-qubit state has five non trivial components in the decomposition (4). As each class has at most four components, we rather consider the following quantity

(33) $$\Lambda_{i}\left(\beta \right)=\max_{|\varphi \rangle ,U_{\mathrm{local}}}\left\{|\langle \varphi \right|U_{\mathrm{local}}^{†}{\left|\beta \rangle \right|}^{2}:|\varphi \rangle \in \mathrm{Class}\;i\},$$

where i={1,2a,2b,3a,3b,4a,4b,4c,4d} and $U_{\mathrm{local}}=U_{1}⊗U_{2}⊗U_{3}$. Such quantity provides an information, how much a given state |β〉 on $H^{⊗3}$ differs from the closest state |φ〉 in the Acín entanglement class *i* [15]. Note that the quantity $\Lambda_{i}$ can be interpreted as the maximal fidelity of a given state |β〉 with respect to the closest state belonging to the class *i*. In particular, if i=1 the results are consistent with $S_{\infty }^{RIU}\left(\beta \right)$ (see [35]) as this yields the maximum overlap with the closest separable state.

By taking a set of $10^{5}$ random states in $H^{⊗3}$, we get their projection $\Lambda_{i}$ on each Acín class, tracking their hyperdeterminant Hdet(|φ〉), which is clearly invariant under local transformations. Then we perform a numerical optimization on the three parameters depicting a local transformation on each qubit (nine in total) together with the necessary coefficients depicting an arbitrary state in each class [15]. Finally, we also track the hyperdeterminant of such a state, Hdet(|β〉). With this information, we construct the corresponding distribution $\rho (\Lambda_{i})$ of each projection *i* (33).

**Figure 7:**
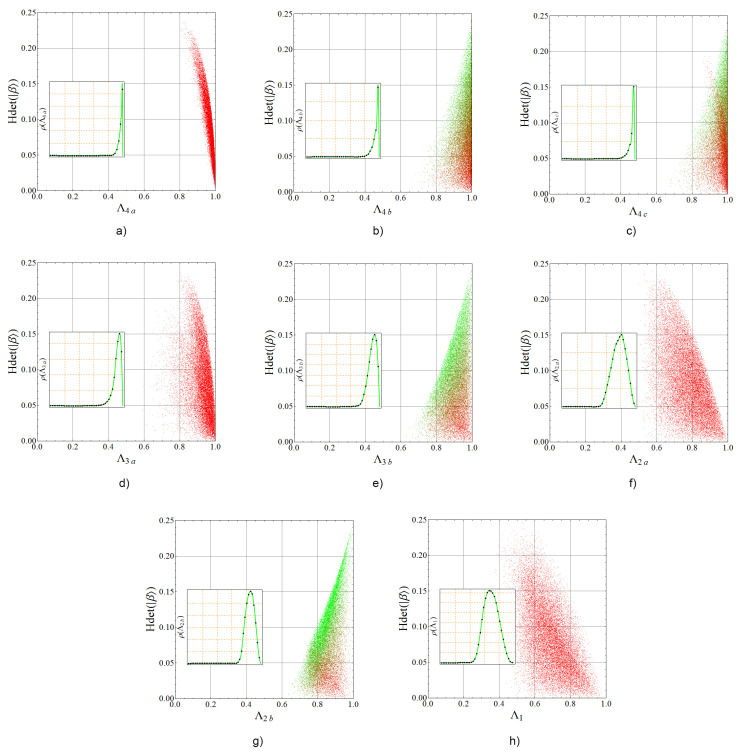
Dispersion graphs showing Hdet(|β〉) versus the maximum overlap $\Lambda_{i}$, colored from red (separable) to green (maximal genuine entanglement). Each panel correspond to one of the Acín classes (see Table 2) as follows: (**a**) Class 4a; (**b**) Class 4b; (**c**) Class 4c; (**d**) Class 3a; (**e**) Class 3b; (**f**) Class 2a; (**g**) Class 2b; (**h**) Class 1. Besides, probability distributions of the maximum overlap (33) are shown in the inset of each plot (vertical scale on the left). We have taken an ensemble of $10^{5}$ three-qubit random states. Graphs of classes 4c and 4d are equivalent so this last was omitted (see details in the core text).

Numerical results are shown jointly in Figure 7. First, the line plot shows the value of $\rho (\Lambda_{i})$ on the left axis versus the value of projection $\Lambda_{i}$ on the horizontal axis. Superposed, a dispersion plot of the entire set of states being analyzed is shown in color. Each dot represents a random state located vertically on their projection value $\Lambda_{i}$ and horizontally in its hyperdeterminant value Hdet(|β〉), which remains invariant under the local optimization procedure. Additionally, each dot is colored in agreement with the hyperdeterminant of the best class element |φ〉 obtained in the optimization. Colors are assigned from red for separable states to green for maximal genuine entanglement. This structure of the plot allows one to compare the closeness between |β〉 and |φ〉 in terms of genuine entanglement. Note the graph corresponding to class 4d has been omitted because it is equivalent to that of class 4c: All coefficients in the class are real, then by exchanging 0 and 1 in all qubits and swapping the qubits 1 and 3 we get the same state with local operations. Thus, the maximal overlap and the hyperdeterminant statistics do not change.

Note particularly how in the Figure 7a the closest class states have Hdet(|φ〉)=0 for some random states which have Hdet(|β〉) near from the highest value $\frac{1}{4}$ maintaining a closer distance $\Lambda_{4a}\approx 1$. The opposite phenomenon is also observed in Figure 7b,c,e,g where some class states with $\mathrm{Hdet}(|\varphi \rangle )\approx \frac{1}{4}$ (in green) are close to some random states with lower Hdet(|β〉) values. On the other hand, in Reference [24] the distribution of the fidelity between two random states has been computed analytically. However, in our case the problem becomes more complicated due to the optimization of the fidelity over all local unitaries.

## 5. The Entanglement Polytope of Three Qubits

Let $\lambda_{k}^{\min}$ denote the smallest eigenvalue of the reduced density matrix of the subsystem of three qubits, where k=A,B,C. The following set of compatibility conditions

(34) $$\lambda_{A}^{\min}\leq \lambda_{B}^{\min}+\lambda_{C}^{\min},\;\lambda_{B}^{\min}\leq \lambda_{A}^{\min}+\lambda_{C}^{\min},\;\lambda_{C}^{\min}\leq \lambda_{A}^{\min}+\lambda_{B}^{\min}.$$

Form particular examples of polygon inequalities obtained by Higuchi et al. for systems of several qubits [17]. The smaller eigenvalue of a one-qubit system is not larger then 1/2 so that $0\leq \lambda_{k}^{\min}\leq 1/2$. Inequalities (34) determine jointly a convex polytope in the three-space $\left(\lambda_{A}^{\min},\lambda_{B}^{\min},\lambda_{C}^{\min}\right)$. Its five vertices represent distinguished three-qubit states: Fully separable states are identified by the point SEP=(0,0,0) whereas points A=(1/2,1/2,0), B=(1/2,0,1/2) and C=(0,1/2,1/2) stand for bi-separable states. The GHZ-state is located at GHZ=(1/2,1/2,1/2). The convex hull of these points is known as the Kirwan polytope [21,47,48]. In addition, the identification of a state belonging to an entanglement classes reported in [20] is summarized in Table 2.

Consider now an ensemble of three-qubit random states. For such states, the probability distribution of the minimal eigenvalue of a single-particle reduced density matrix fulfils $P\left(\lambda_{\min}\right)=P\left(\lambda_{A}^{\min}\right)=P\left(\lambda_{B}^{\min}\right)=P\left(\lambda_{C}^{\min}\right)$. Using the following relation between the two eigenvalues $\vartheta_{1}$ and $\vartheta_{2}$ of a single qubit reduced density matrix

$$\lambda_{\min}=\min\left(\vartheta_{1},\vartheta_{2}\right)=\frac{1}{2}\left(\vartheta_{1}+\vartheta_{2}\right)-\frac{1}{2}\left|\vartheta_{1}-\vartheta_{2}\right|,$$

we can compute the probability distribution of the minimal eigenvalue $\lambda_{\min}$ as

(35) $$P\left(\lambda_{\min}\right)=\int_{0}^{1}\int_{0}^{1}d\vartheta_{1}d\vartheta_{2}P\left(\vartheta_{1},\vartheta_{2}\right)\delta [\lambda_{\min}-\left(\vartheta_{1}+\vartheta_{2}\right)/2+|\vartheta_{1}-\vartheta_{2}|/2)],$$

where $P(\vartheta_{1},\vartheta_{2})$ is the joint density (7) and δ stands for the Dirac delta function. Performing the integral, we obtain

(36) $$P\left(\lambda_{\min}\right)=420{\left[\lambda_{\min}\left(2\lambda_{\min}-1\right)\left(1-\lambda_{\min}\right)\right]}^{2},\;0\leq \lambda_{\min}\leq 1/2.$$

**Figure 8:**
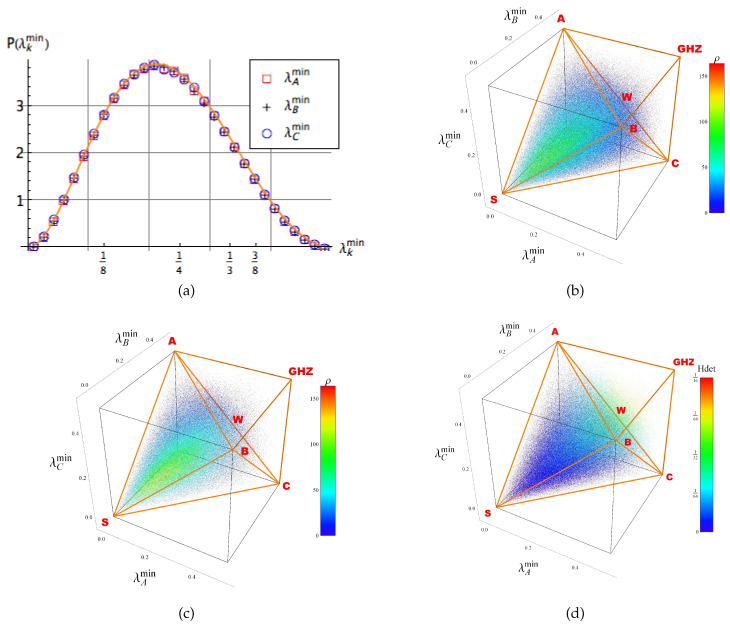
(**a**) Probability distribution of the minimal eigenvalue of a single qubit reduced system (36). (**b**) An ensemble $10^{6}$ of three-qubit random states depicted in the entanglement polytope. The color scale stands for the joint probability distribution. (**c**) Detail of (**b**): A transversal section by the plane which contains the points *S*, *C* and GHZ. (**d**) The ensemble of three qubit random states labeled by colour settled according to the value of the invariant $I_{6}$.

This distribution is depicted in Figure 8. Besides, a direct calculation yields the average value $\langle \lambda_{\min}\rangle =29/128$. In general, the *k*-the moment of $\lambda_{\min}$ reads

(37) $$\langle \lambda_{\min}^{k}\rangle =\frac{105}{2^{k}}\left[\frac{\Gamma (k+3)}{\Gamma (k+6)}-\frac{\Gamma (k+4)}{\Gamma (k+7)}+\frac{\Gamma (k+5)}{4\Gamma (k+8)}\right].$$

Note that a given pure state can be identified with a point in the entanglement polytope. Its coordinates are $\left(\lambda_{A}^{\min},\lambda_{B}^{\min},\lambda_{C}^{\min}\right)$. This is shown in Figure 8b for an ensemble of $10^{6}$ three-qubit random states colored according to their joint probability distribution in the polytope. To compute such probability distribution, the space containing the whole polytope ${\left[0,\frac{1}{2}\right]}^{\times 3}$ was divided into $80^{3}$ cubic cells. Then, we state the statistics of random states falling in each cell to get the probability density of those states (by volume unity). Note that the closer the points are to the faces, the lower the value of the distribution. In Figure 8c we depict a transverse cut by the plane containing the vertices *S*, *C* and GHZ to depict the distribution of the inner points. This shows that random states are more concentrated near the line joining the vertices SEP and GHZ, which corresponds to class 2a.

On the other hand, two quantum pure states attain the same amount of entanglement if they belong to the same class, that is to say if there is a finite probability of success that they can be converted into each other using stochastic local operations and classical communication, referred to as SLOCC by its acronyms. For the case of three qubits, there exist two SLOCC classes of entanglement: the one containing the GHZ state, which exhibits genuine entanglement and the *W* class [8].These classes can be distinguished from the entanglement polytope. Numerical calculation shows that around 6% of the states are placed in the upper polytope, so that they belong to the GHZ SLOCC class [21]. As the invariant $I_{6}$ discriminates between such classes in panel Figure 8d we show the ensemble of random states colored with respect to this invariant. For states placed near the bi-separable faces $I_{6}$ goes to zero, whereas the states landing in the GHZ simplex are characterized by a positive value of this invariant. An equivalent approach can be done dealing with the maximum eigenvalues of the reduced single qubit density matrices. For such a case, the joint probability distribution is known [49] and hence the fraction of random states in the GHZ pyramid was computed in Reference [50] yielding 13/216≈6.02% which is consistent with our numerical calculation.

## 6. Conclusions and Future Work

We studied various quantities describing a three-qubit pure quantum state and analyzed their probability distributions obtained for an ensemble of random pure states generated by the unitary invariant Haar measure. In particular, we investigated the distribution of the six parameters determining the five-terms decomposition (4) of a three-qubit state. The phase of the complex coefficient occurs to be uniformly distributed. The distributions of the amplitudes $\lambda_{0}$ and $\lambda_{4}$ differ from the distribution describing the remaining three coefficients. Interestingly, these two coefficients can be related with the degree of entanglement as the invariant $J_{4}$ depends only on them. In addition, we have also analyzed the probability distributions of two sets of polynomial invariants. The invariants $I_{1},I_{2}$ and $I_{3}$ follow the same distribution. Thus, out of the five independent invariants, only three are necessary to characterize entanglement in three-qubit states. This fact is consistent with the second set of invariants reported by Acín et al. as the distributions of the invariants $J_{1},J_{2}$ and $J_{3}$ do coincide. For each invariant its mean value was computed using symbolic integration with respect to the unitary invariant Haar measure. Moreover, we have also obtained the probability distribution of the anisotropic strength $s_{\mathrm{ani}}$ and the anisotropic volume $V_{\mathrm{ani}}$ introduced recently in [37]. These invariants are useful in the study of strong monogamy relations, geometric discord and fidelity of remote state preparation and studies of violation of the Bell inequality. In this last context, one could ask for the probability that one of the three pairs violates a Bell inequality. However, these results will be reported elsewhere.

On the other hand, the set of invariants $\left\{J_{k}\right\}$ allows us to identify certain entanglement classes, whose entanglement was described through the minimal decomposition entropy. Moreover, highly entangled states with respect to this measure were identified in each class. Our results imply that the more terms in the decomposition (4) of a three-qubit state, the larger its degree of entanglement measured by the minimal decomposition entropy.

The numerical outcomes provide us with several insights about possible meanings of the entanglement invariants. First, there is an apparent underlying statistical equivalence between coefficients $\lambda_{1},\lambda_{2}$ and $\lambda_{3}$ (and their low values suggest a closer position of the states respecting genuine entanglement states in terms of the RIU entropy statistics for the overall 3-qubits random states). The same aspects seems true for $I_{2},I_{3},I_{4}$ and $J_{1},J_{2},J_{3}$ invariants. Together, larger values for $\lambda_{0}$ and low values for $I_{5}^{\prime \prime \prime }$ and $J_{4}$ seem related with the presence of genuine entanglement (this affirmation is based on the fact that larger values of RIU entropy are statistically more common for the overall 3-qubit random states).

Other outcomes relative to the type a in the Acín classes exhibit separable states. There, the growing number of the class (1,2,…,4) reflects the inclusion of most of the random states for three qubits (see Figure 7a,d,f,h). In this sense, the use of RIU entropy as an exemplary measure of quantum entanglement allows us to provide a classification of three qubit states and to describe their hierarchy. The invariants with respect to local transformations are useful to identify certain types of entangled structures in the entire system. As shown in Figure 7, the states displaying genuine entanglement appear closer from other states in the classes with no genuine entanglement. Although smooth measures of entanglement depend on the state in a continuous way, a small variation of a state can lead to a considerable change of its entanglement. This feature was observed in larger systems [51]. In such a scenario, the current analysis in the quest of understanding the hierarchy of entanglement, could set directions to transform states from maximally entangled into separable ones. By using the SU(2) decomposition procedure, [52] has been clear about the existence of basic U(1)×SU(2) operations among entangled pairs, showing how the entanglement phenomena can be generated in a structured way form basic operations then transiting from separable to genuine entangled states. This suggests that programmed local operations combined with entangling operations between two previous entangled pairs can be realized in order to connect such state types. Thus, basic separable states could be transformed into maximal entangled states as |GHZ〉 and |W〉 only with a series of such operations. In a more ambitious task, those single types of operations could suggest they could be responsible for the transit from certain classes to others among the hierarchies of entanglement. In such a process, the track in the change of the invariants values could provide a strong road-map for such transit.

Finally, we have analyzed the probability distribution of the maximal fidelity of a random state with respect to the closest representative of each entanglement class. The highest maximal fidelity is obtained for classes 4a–d listed in Table 2. This can be seen from the fact that the distributions of five coefficients in the decomposition (4) are highly non-trivial, as these quantities carry some information concerning the degree of entanglement. Our study comprises several ways to analyze the entanglement in a three-qubit system showing the fact that entanglement can be characterized from different approaches, each one providing different aspects of non-locality. Therefore, we hope the results of this work will shed some light on the matter.

## Figures and Tables

**Table 1:** Best numerical fit parameters of the distributions $P_{i}\left(\lambda_{i}\right)=c\;\lambda_{i}^{a}{\left(1-\lambda_{i}\right)}^{b}$ for i=0,1,2,3,4.

i	a	b	c
0	3.74	6.05	1856.85
1	67.76	4.25	1.52
2	68.40	4.27	1.53
3	66.75	4.24	1.52
4	795.16	4.37	3.96

**Table 2:** Acín entanglement classes introduced in [15]. Besides $\Delta_{J}\equiv {\left(J_{4}+J_{5}\right)}^{2}-4\left(J_{1}+J_{4}\right)\left(J_{2}+J_{4}\right)\left(J_{3}+J_{4}\right)$. Basis elements marked with ⋆ are not directly obtained, instead they have additional relabellings. Besides, the fourth column shows the identification of each class with subsets of the entanglement polytope. The point *G* stands for (1/2,1/2,1/2). Details are presented in Section 5.

Class	Conditions	States	Entanglement Polytope
1	$J_{i}=0$	|000〉	point O=(0,0,0)
2a	All $J_{i}=0$ apart from $J_{1}$	${|000\rangle ,|011\rangle }^{⋆}$	lines $\bar{OA}$, $\bar{OB}$ and $\bar{OC}$
2b	All $J_{i}=0$ apart from $J_{4}$	|000〉,|111〉	line $\bar{OG}$
3a	$J_{1}J_{2}+J_{1}J_{3}+J_{2}J_{3}=\sqrt{J_{1}J_{2}J_{3}}=J_{5}/2,\;J_{4}=0$	|000〉,|101〉,|110〉	${▵}_{2}OAB$, ${▵}_{2}OAC$, ${▵}_{2}OBC$, ${▵}_{2}ABC$
3b	$J_{1}=J_{2}=J_{5}=0$	|000〉,|110〉,|111〉	${▵}_{2}ABG$, ${▵}_{2}ACG$, ${▵}_{2}BCG$
4a	$J_{4}=0,\sqrt{J_{1}J_{2}J_{3}}=J_{5}/2$	|000〉,|100〉,|101〉,|110〉	${▵}_{3}OABC$
4b	$J_{2}=J_{5}=0$	|000〉,|100〉,|110〉,|111〉	
4c	$J_{1}J_{4}+J_{1}J_{2}+J_{1}J_{3}+J_{2}J_{3}=\sqrt{J_{1}J_{2}J_{3}}=J_{5}/2$	|000〉,|101〉,|110〉,|111〉	
4d	$\Delta_{J}=0,\sqrt{J_{1}J_{2}J_{3}}=\left|J_{5}\right|/2$	${|000\rangle ,|010\rangle ,|100\rangle ,|111\rangle }^{⋆}$	
